# Case report: Precision guided reactive cancer management: molecular complete response in heavily pretreated metastatic CRC by dual immunotherapy and sorafenib

**DOI:** 10.3389/fonc.2024.1405170

**Published:** 2024-07-01

**Authors:** Esranur Aydın, Ünal Metin Tokat, Ashkan Adibi, Eylül Özgü, Şevval Nur Bilgiç, Mutlu Demiray

**Affiliations:** ^1^ Precision Oncology Center, Medicana Health Group, Istanbul, Türkiye; ^2^ Department of Basic Oncology, Division of Cancer Genetics, Institute of Oncology, University of Istanbul, Istanbul, Türkiye

**Keywords:** colorectal cancer, FLT3, high TMB, dual immunotherapy, sorafenib, case report, MRD, precision oncology

## Abstract

**Background:**

Metastatic colon adenocarcinoma presents significant challenges in treatment, particularly when resistant to standard therapies. Precision oncology, guided by multidisciplinary tumor boards (MTBs), offers a promising way for individualized therapeutic approaches. Integration of comprehensive genomic profiling (CGP) and minimal residual disease (MRD) testing strengthens treatment decision-making, yet challenges persist in identifying and overcoming resistance mechanisms. FLT3 amplification can be one of those resistance/escape mechanisms that needs to be targeted.

**Case presentation:**

This case report presents a 58-year-old male diagnosed with metastatic colon adenocarcinoma with liver metastasis, resistant to conventional treatments. Utilizing CGP and MRD testing, our multidisciplinary MTB identified a complex mutational profile, including APC, DAXX, TP53 mutations, and CDK8 and FLT3 amplifications. With a tumor mutational burden of 10 muts/mb and TPS, CPS scores of 0, immunotherapy was considered, employing dual immune checkpoint inhibitors alongside mebendazole and Lenvatinib targeting the WNT and VEGF/angiogenesis pathways. MRD testing revealed early treatment failure. Re-evaluation identified high copied FLT3 amplification (62 copies) as a resistance mechanism, prompting modification to incorporate sorafenib and dual immunotherapy with mebendazole. Subsequent MRD assessments and radiological scans demonstrated a remarkable therapeutic response, with sustained efficacy and absence of detectable residual disease.

**Conclusion:**

This case highlights the successful application of precision oncology principles, facilitated by dynamic MTB-guided treatment strategies. Integration of MRD testing provided early detection of treatment inefficacy, allowing for timely intervention and adaptation of the treatment plan. Additionally, the case highlights the educational value of rare molecular alterations, emphasizing continual learning and refinement of treatment approaches in precision oncology.

## Introduction

Colorectal cancer (CRC) remains a major global health concern, necessitating ongoing therapeutic advancements to address its heterogeneity and resistance to conventional therapies. The standard of care for metastatic colorectal cancer (mCRC) traditionally involves a combination of surgery, chemotherapy regimens, and targeted therapies. Surgery is crucial for early-stage CRC, potentially curative when localized, and in mCRC, it can resect metastases to prolong survival and improve quality of life.

Chemotherapy regimens such as FOLFOX (folinic acid, fluorouracil, and oxaliplatin) or FOLFIRI (folinic acid, fluorouracil, and irinotecan) are often combined with targeted therapies like bevacizumab or anti-EGFR antibodies (cetuximab and panitumumab) for patients with wild-type RAS tumors. Despite advances in these treatments, the prognosis for mCRC remains poor, with a five-year survival rate of approximately 15% (1). This striking statistic underscores the urgency for developing more effective treatment strategies and the need for continuous innovation in therapeutic approaches.

A significant challenge in the management of mCRC is the development of resistance to these conventional therapies. Chemotherapy resistance can occur through various mechanisms, including drug efflux, enhanced DNA repair, and evasion of apoptosis. For instance, alterations in the tumor microenvironment, such as hypoxia and stromal interactions, can promote a survival advantage for cancer cells under therapeutic pressure (2). Additionally, mutations in key oncogenes and tumor suppressor genes can confer resistance to targeted therapies. For example, secondary mutations in the EGFR pathway or KRAS gene can lead to resistance against anti-EGFR therapies (3).

Immunotherapy has emerged as a promising option for a subset of CRC patients, particularly those with microsatellite instability-high (MSI-H) or mismatch repair deficient (dMMR) tumors (4). Immune checkpoint inhibitors, such as pembrolizumab and nivolumab, have shown significant efficacy in this population, offering new hope for improved outcomes. However, the majority of CRC patients do not benefit from immunotherapy, highlighting the need for novel approaches to identify and overcome resistance mechanisms. Mechanisms of resistance to immunotherapy can include loss of neoantigen expression, alterations in immune checkpoint molecule expression, and changes in the tumor microenvironment that suppress immune cell infiltration.

Survival outcomes in CRC vary significantly based on demographic factors, including gender. Studies show that median overall survival (OS) for patients with mCRC is influenced by these variables. For instance, young women with CRC have a higher survival rate than men of the same age group (5). SEER Database analysis reveals a disparity in median OS for mCRC based on age at diagnosis. Patients with early-onset mCRC (diagnosed before age 50) have a median OS of approximately 30 months, compared to 18 months for those with average-onset mCRC (6).

In the landscape of cancer care, precision oncology emerges as a transformative approach, tailoring disease management to the unique molecular profiles of tumors. This shift has led to the growth of Molecular Tumor Boards (MTBs) as pivotal platforms for collaborative decision-making, particularly in complex cases. MTBs facilitate the integration of multidisciplinary expertise, ensuring that each patient’s treatment plan is informed by the latest scientific insights and tailored to their specific genetic and molecular tumor characteristics.

At the heart of precision oncology lies Comprehensive Genomic Profiling (CGP), which plays a fundamental role in providing a detailed assessment of the genetic landscape of colorectal tumors. By analyzing multiple genes simultaneously, CGP enables the identification of actionable mutations and potential resistance mechanisms, guiding treatment decisions toward more effective and personalized therapeutic interventions. This empowers clinicians to choose targeted treatments that directly address the molecular abnormalities driving the cancer, optimizing outcomes while reducing unnecessary exposure to ineffective treatments.

Moreover, CGP goes beyond mere analysis; it serves as a tool for unraveling the complex genetic characteristics of colorectal tumors, providing clinicians with invaluable insights that inform targeted treatment approaches. Addressing rare and uncommon molecular alterations requires a science-driven, innovative approach to improve survival outcomes, emphasizing continuous learning and reactive decision making in clinical management.

Here, we present a compelling case of a 58-year-old male with metastatic CRC resistant to standard therapies. CGP revealed a complex mutational profile, including high copy numbered (62 Copies) FLT3 amplification, implicating its potential role in resistance mechanisms. Through the integration of multidisciplinary MTBs, CGP, and minimal residual disease (MRD) monitoring, we identified a complex mutational profile, including APC, DAXX, TP53 mutations, and CDK8 and FLT3 amplifications. Treatment initially involved immunotherapy alongside targeted therapies, with subsequent modifications guided by MRD testing results, leading to a remarkable therapeutic response and sustained efficacy.

**Figure 1:**
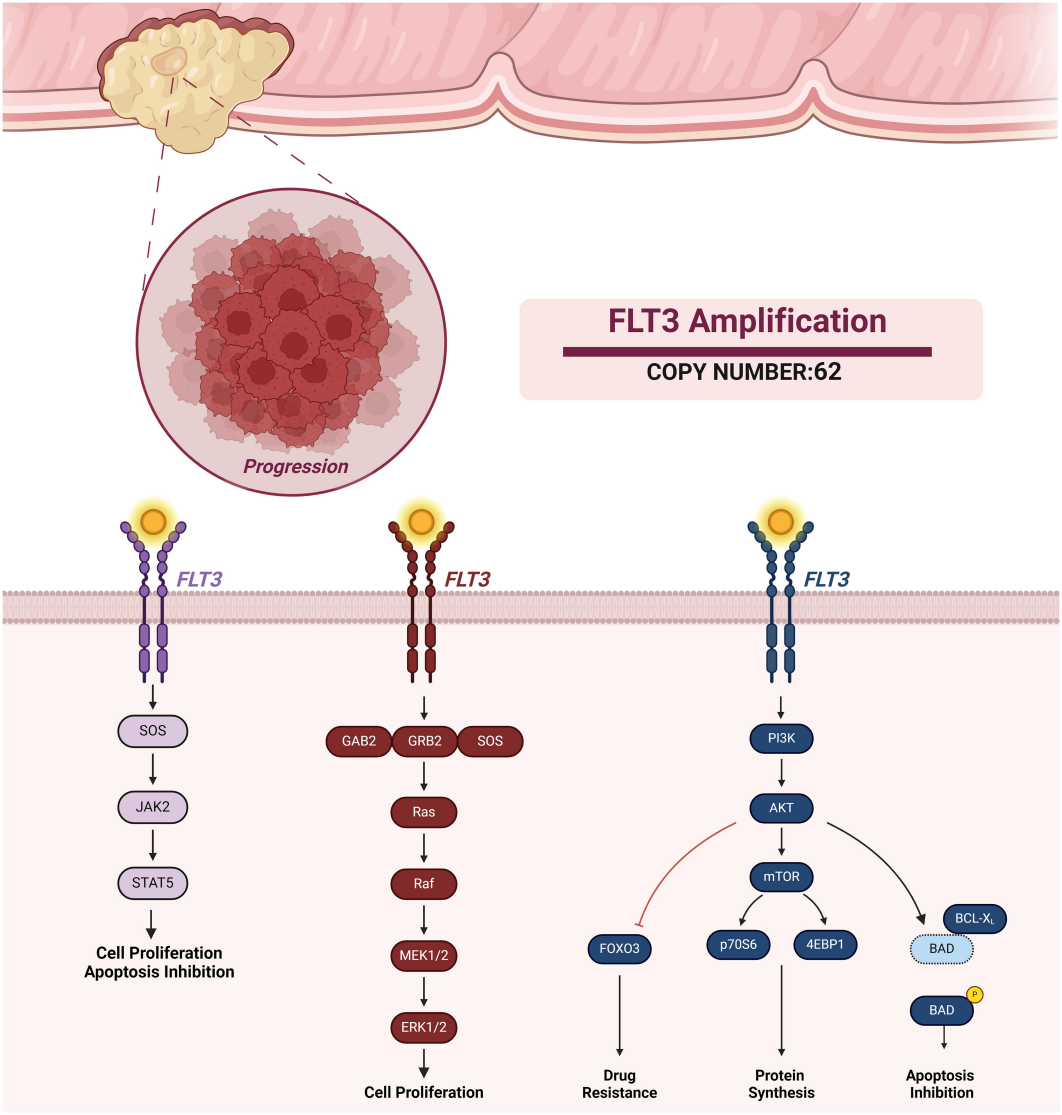
FLT Amplification and Associated Signaling Pathways. FLT3 activation promotes different pathways such as JAK-STAT, MAPK, and PI3K signaling. FLT3 activation of these signaling pathways results in cell proliferation, apoptosis inhibition, protein synthesis, and drug resistance.

Notably, our analysis revealed a high copy number FLT3 amplification (62 Copies) in the patient’s mutational profile. The FLT3 gene encodes a receptor tyrosine kinase critical for regulating hematopoietic stem and progenitor cell growth and differentiation. When activated by its ligand, FLT3 triggers signaling cascades involving pathways such as the MAPK, PI3K/AKT, and STAT, ultimately regulating cell proliferation, survival, and differentiation (7) as shown in **Figure 1**. While FLT3 expression or mutations are more commonly associated with hematological malignancies, emerging evidence suggests their potential roles in solid tumors, including CRC. However, further research is needed to fully elucidate the significance of FLT3 in solid cancers.

As precision oncology continues to redefine the cancer treatment landscape, this case offers a convincing example of its successful application, emphasizing the need for adaptability and continuous learning. The lessons derived from this case contribute not only to the refinement of treatment strategies for patients with unique molecular profiles but also serve as a valuable reference for clinicians and researchers engaged in the evolving landscape of precision oncology.

## Case presentation

Here we present a 58-year-old male diagnosed with metastatic colon adenocarcinoma, accompanied by a single liver metastasis at the time of initial diagnosis. The patient’s medical history revealed that multiple conventional treatments initiated at another clinic have ultimately failed. The patient initially underwent FOLFOX chemotherapy alongside being operated on primer tumor and having a metastasectomy. Upon disease progression with new liver metastasis, the treatment was shifted to FOLFIRI, accompanied by radiofrequency ablation (RFA) on the metastatic sites. Despite these efforts, further progression necessitated a second round of RFA, and the chemotherapy regimen was modified to FOLFIRINOX plus cetuximab for the 3^rd^ line. After three cycles of the new regimen, the patient once again experienced disease progression with liver metastasis, leading to his visit to our precision oncology center for a second opinion.

Following the Radiofrequency Ablation (RFA), the patient underwent monitoring via tumor informed MRD testing by Signatera™. Despite the absence of radiologically detected tumors, the initial MRD test revealed a value of 67.52 MTM/mL. Consequently, our MTB recommended a systemic treatment followed by a CGP test to unravel the mutational profile of the tumor and design the most appropriate treatment plan. The genomic profiling revealed a complex mutational profile, including APC, DAXX, and TP53 mutations accompanied by CDK8 and FLT3 amplifications. Notably, the patient exhibited a tumor mutational burden (TMB) of 10 muts/mb with a negative PD-L1 IHC (**Table 1**).

**Table 1:** The CGP profile of the patient.

FoundationOne CDx NGS Results		
Tumor Mutation Burden	10 muts/mb	
Microsatellite Status	MS Stable	
Gene	Alteration	VAF % or Copy Number
**APC**	E1309fs*4	90.5%
**CDK8**	amplification	8 copies
**DAXX**	S588F	25.4%
**FLT3**	amplification	62 copies
**TP53**	C124fs*25	93.7%

**Figure 2:**
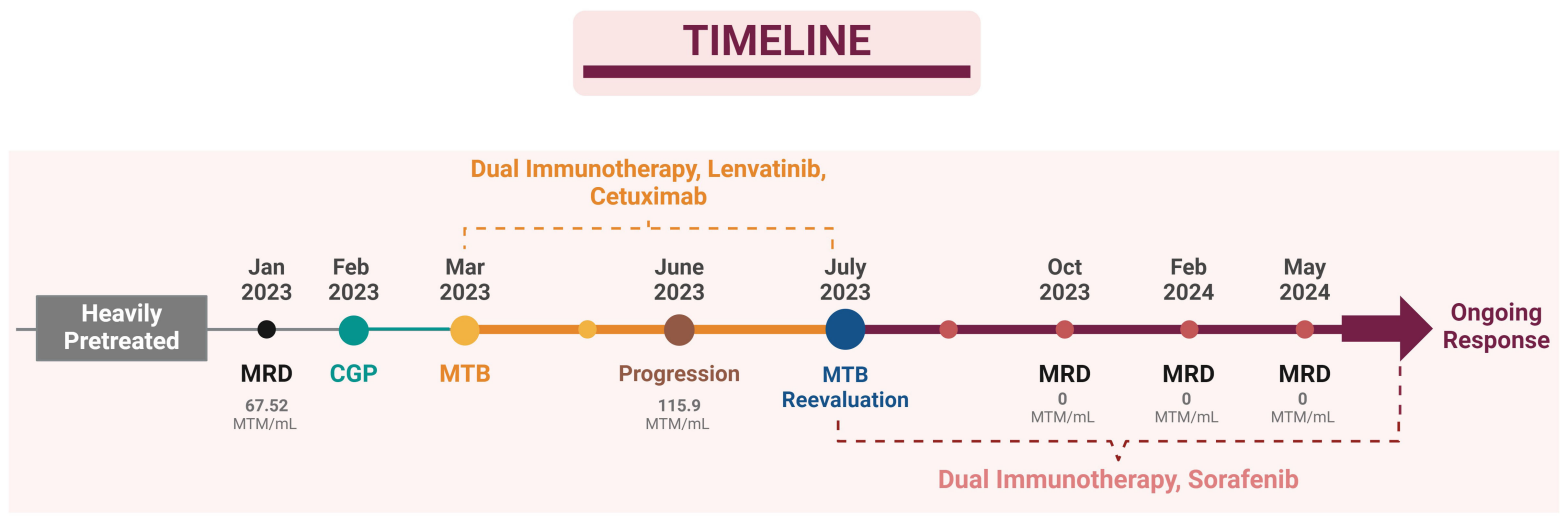
Patient’s Treatment Timeline.

With the patient displaying a TMB of 10 muts/mb, the MTB considered immunotherapy as a viable treatment option. Provided that multiple immune checkpoint proteins on tumor cells were expressed at low levels, the MTB recognized the potential limitations of single-agent immunotherapy and opted for a dual immunotherapy approach (**Figure 2**). This strategy utilizes two distinct immune checkpoint inhibitors (ICIs): an anti-PD-1 and an anti-CTLA4 antibody. The rationale behind this approach is to elicit a more potent antitumor immune response and potentially overcome resistance mechanisms that may arise with a single-agent ICI therapy. Additionally, the presence of loss-of-function APC and TP53 alterations prompted the incorporation of mebendazole and Lenvatinib targeting the WNT and VEGF/angiogenesis pathways respectively. Nivolumab was given at a monthly dose of 400 mg, ipilumab was administered every two months at a dose of 50 mg, lenvatinib was prescribed at a daily dosage of 10 mg, and mebendazole was used twice daily at a dose of 100 mg each time.

The patient’s treatment response was monitored through serial MRD testing and radiological scans. The first MRD, performed three weeks post-RFA, revealed a result of 67.52 MTM/mL. Treatment started at the beginning of March, and early signs of treatment ineffectiveness were observed after the second MRD test and radiological scanning during the third-month checkup. MRD testing reported an unexpected increase to 115.9 MTM/mL, indicating the progression of disease despite the treatment regimen. Subsequent imaging revealed tumor growth around previous RFA sites and the emergence of two new lesions. Limited radioembolization was planned for these lesions and MTB re-evaluated the molecular profile of the patient for the new treatment plan since he did not respond to the designed treatment from the beginning. FLT3 amplification was identified as the possible resistance/escape mechanism. Initially overlooked due to its rarity in solid tumors, especially colon cancers, this alteration underscored the importance of continuous and reactive treatment guidance in the era of precision oncology MTBs. The copy number profile revealed a copy number of 62 copies of the *FLT3* gene. FLT3 inhibitors, midostaurin, and sorafenib were evaluated. Sorafenib, targeting FLT3 and the VEGF pathway, was prioritized over midostaurin due to its broader spectrum of activity and immunomodulatory effects.

**Figure 3:**
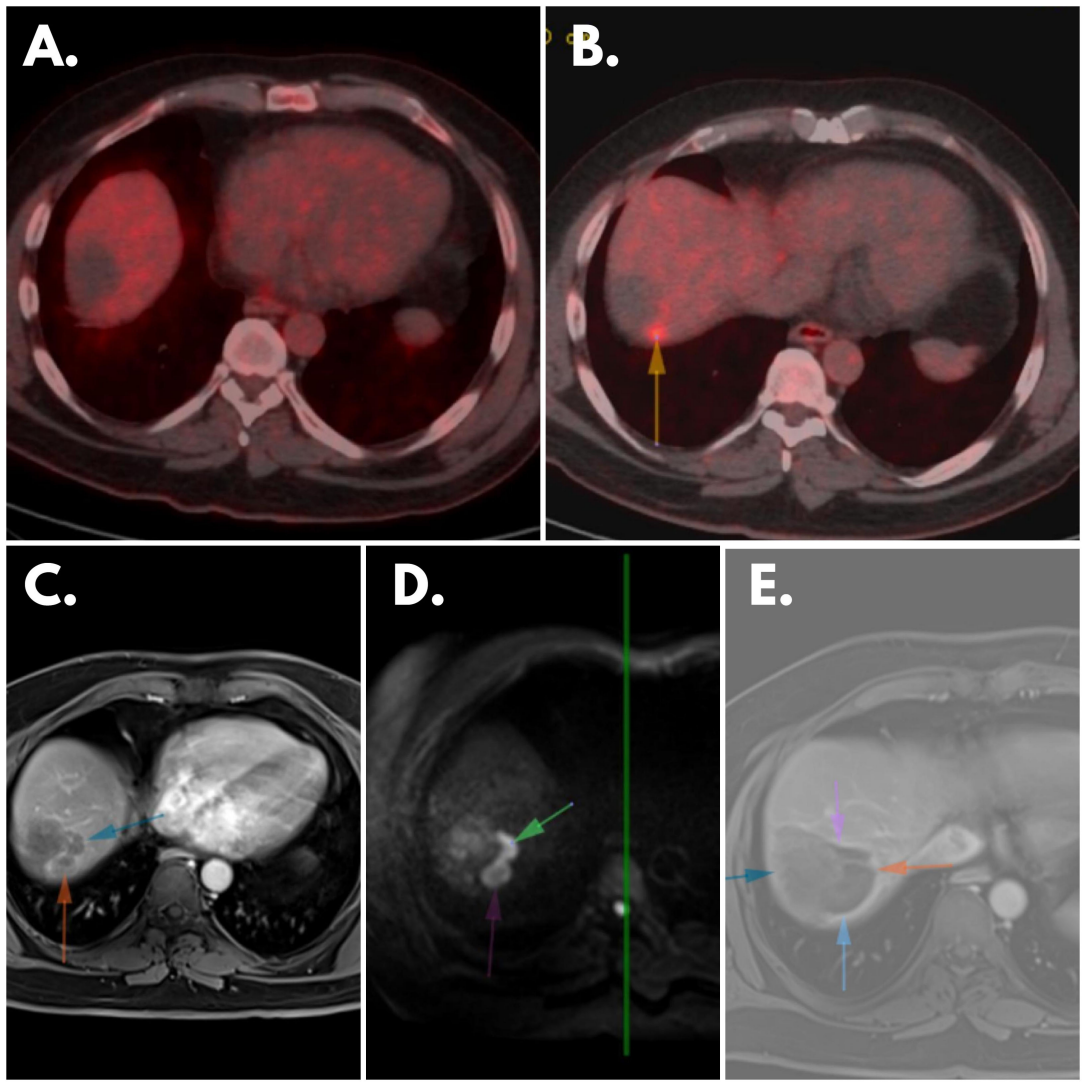
Patient’s Scannings **(A)** February 2023 PET: No significant FDG accumulation observed in the area of the hypodense lesion indicating lack of metabolic activity. **(B)** May 2023 PET: Newly developed focal FDG uptake focus (arrow) evident in immediate posterosuperior neighborhood of previously identified lesion, suggesting change in metabolic activity, indicative of disease progression. **(C)** May 2023 MR Scanning: Peripheral contrast-enhancing lesions visible in localization described in February 2023’s PET, suggesting presence of enhanced vascularity. **(D)** May 2023 MR Scanning: Diffusion-restricting lesions observed in same localization, indicating restricted water diffusion within lesions, potential marker of cellular density and tumor activity. **(E)** Post-treatment MRI: Cavitary area without contrast enhancement and diffusion restriction evident in lung, suggesting treatment-induced change such as necrosis or resolution of previously identified lesion, signifying favorable treatment response in this specific anatomical area.

The treatment plan was revised to incorporate dual immunotherapy with sorafenib and mebendazole, with simultaneous RFA performed on the identified lesions. Sorafenib was prescribed at a daily dosage of 200 mg, while dual immunotherapy and mebendazole were maintained at their previous dosages. The following MRD test, conducted three months after this modified treatment regimen and post-treatment radiological scanning (**Figure 3**) both revealed a remarkable therapeutic response with a drastic reduction to zero at the MRD test (**Table 2**), signifying a substantial and favorable treatment outcome. Subsequent MRD evaluations, performed in February 2024 and May 2024, corroborated the sustained efficacy of the treatment, demonstrating continued absence of detectable residual disease.

**Table 2:** Minimal residual disease follow-up of the patient.

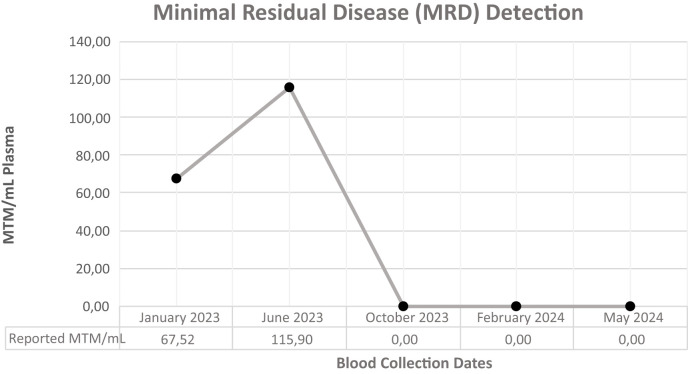

## Discussion

The presented case illustrates the complexity and challenges inherent in the management of advanced colon adenocarcinoma with liver metastasis, particularly due to treatment resistance and the need for continuous disease monitoring. The patient’s initial management at another clinic involved a series of conventional irinotecan and oxaliplatin based chemotherapy regimens. Additionally, surgical interventions were undertaken to address the primary tumor and metastatic lesions. Furthermore, local treatment modalities like the RFA and radioembolization were employed for the metastatic sites; however, the disease continued to progress. Seeking a second opinion, the patient turned to our precision oncology center, where the CGP was recommended, and the case was discussed in our molecular tumor board.

In the context of CRC management, the utilization of the MRD assessment has emerged as a critical tool in response monitoring and clinical decision making. Traditionally, adjuvant chemotherapy in CRC has been determined primarily by clinicopathologic factors, which, while important, may not comprehensively reflect the patient’s individual response or prognosis. In our clinical practice, we have integrated MRD assessment as a critical component for adjuvant therapy decisions in CRC patients (8).

Bringing MRD monitoring into the practice enables the detection of residual disease at levels beyond the reach of standard imaging techniques. This real-time feedback mechanism, as demonstrated in this case, proves crucial in the early identification of treatment inefficacy, enabling timely interventions and adjustments to the therapeutic approach. In this particular case, the initial MRD measurement of 67.52 MTM/mL, despite local treatment and the absence of radiologically detected lesions, prompted the initiation of treatment. However, despite the intervention, subsequent MRD assessment revealed a concerning increase to 115.9 MTM/mL, indicative of treatment ineffectiveness. This highlights the dynamic nature of CRC and the importance of serial MRD testing to accurately monitor treatment response and adjust therapeutic strategies accordingly.

Moving forward, the incorporation of MRD assessment into routine clinical practice holds promise for enhancing the precision of treatment decisions in CRC based on several studies (9). By identifying patients who are most likely to benefit from adjuvant therapy and enabling timely adjustments in treatment regimens, MRD evaluation contributes to a more individualized and efficacious approach to CRC management. Further research and clinical experience are warranted to validate the utility of MRD assessment and optimize its integration into standard care pathways for CRC patients.

In addition to advancements in MRD assessment, immunotherapy has emerged as a transformative modality in the management of cancer, particularly for those harboring tumors with elevated TMB, indicative of increased neoantigen load and potential susceptibility to immune checkpoint blockade. Despite the patient’s TMB exceeding the conventional threshold for TMB-high status, our molecular tumor board adopts a comprehensive approach to treatment decision-making, integrating not only the absolute TMB value but also the patient’s immune microenvironment and the median TMB values observed in CRC studies. As the body of scientific research continues to expand, it becomes increasingly apparent that the current one-size-fits-all TMB threshold of 10 muts/mb requires reevaluation. This necessity arises from the observed variability in TMB levels across distinct cancer types and patient cohorts. Recent investigations into the genetic landscape of CRC underscore the imperative of tailoring TMB thresholds to individual patient profiles, considering the nuanced nature of mutational burdens in CRC. Studies focusing on CRC genetics reveal a notable disparity between the typical TMB levels observed in colon adenocarcinomas and the established cutoff point. Specifically, median TMB values for colon adenocarcinomas fall within the range of 3.68 mutations per megabase (muts/mb) (10) to 4.5 muts/mb (11), markedly lower than the conventional threshold. Furthermore, the distinct characteristics of microsatellite stable (MSS) and microsatellite instability-high (MSI-H) CRC necessitate the development of separate TMB stratification models. For instance, studies have demonstrated median TMB values of 3.6 muts/mb in MSS and a staggering 46.8 muts/mb in MSI-H CRC patients (12), suggesting distinct immunogenic profiles require individualized therapeutic strategies.

Clinical trials like CALGB/SWOG 80405 have proposed alternative TMB cutoffs based on patient stratification and outcomes. In this trial, the TMB threshold for distinguishing low and high TMB was set at 8 muts/mb, reflecting the dynamic nature of TMB classification and its implications for treatment selection (13). In this particular case, the patient’s TMB not only surpassed the tumor-agnostic TMB-high threshold but also exceeded the median TMB levels reported in CRC studies. This led the molecular tumor board to consider immunotherapy as a viable treatment option.

While high TMB typically suggests a potential benefit from immunotherapy, due to the presence of neoantigens, a nuanced examination of tumor proportion score (TPS) and comprehensive score (CPS) uncovered subdued expression levels of immune checkpoint proteins. Concurrent assessment of TPS and CPS revealed low expression of immune checkpoint proteins. Recognizing the potential limitations of single-agent immunotherapy in cases of low TPS and CPS scores, the MTB recommended a dual immunotherapy combination, involving two distinct immune checkpoint inhibitors (ICIs) -anti-PD-1 and anti-CTLA4- to trigger a more robust antitumor immunity and thereby overcoming potential resistance mechanisms to the individual ICIs.

According to studies, tumors with elevated TMB may elicit an immunological response, however, CTLA-4 inhibits T-cell infiltration and activation. This control could be achieved either by an intrinsic increase in CTLA-4 on Teff cells or through regulation by CTLA-4hi Treg cells. Reduced IFN-γ levels in tumors correlate with low or negative PD-L1 status, indicating minimal T-cell activation during diagnosis. However, by inhibiting CTLA-4 or depleting Tregs, Teff cells can be reactivated, resulting in upregulation of PD-1 and consequent PD-L1 production, increasing the efficacy of PD-1 inhibition. Thus, CTLA-4 blockage may enhance the efficacy of PD-L1 blocking, implying that combination immunotherapy may provide improved results (14).

The presence of a loss-of-function APC alteration prompted the repurposing of mebendazole, targeting the Traf2 and Nck-interacting protein kinase (TNIK) downstream of the WNT pathway, adding a unique dimension to the therapeutic intervention. TNIK has garnered attention as a key regulator within the β-catenin and T-cell factor-4 (TCF-4) transcriptional complex which is a critical signaling pathway implicated in cancer development and progression (15). Emerging evidence suggests that modulation of TNIK activity could offer a potential for therapeutic intervention. In recent years, several small molecule compounds targeting TNIK have been investigated for their anti-tumor effects. These compounds exhibit promising results in preclinical studies, demonstrating efficacy against a spectrum of malignancies. By selectively inhibiting TNIK, these agents exert their effects on downstream signaling pathways, disrupting aberrant Wnt signaling and impeding tumor growth and progression. One notable compound that has emerged from this research is Mebendazole, traditionally known as an anthelmintic agent. Mebendazole has been identified as a selective inhibitor of TNIK and it has been evaluated for its anticancer properties. Its mechanism of action involves the regulation of Wnt signaling downstream of the TNIK pathway, offering a targeted approach to modulating this dysregulated signaling cascade (16). A case report documented a patient with metastatic adrenocortical cancer who experienced disease stabilization and tumor shrinkage after Mebendazole monotherapy, following prior treatment failures. Notably, the patient received a standard anti-helminthic dose (100mg twice daily) and achieved 19 months of disease control (17). Similarly, another case report described a patient with advanced metastatic colon cancer who responded remarkably to Mebendazole after conventional chemotherapy failed. Within six weeks of Mebendazole treatment (again at 100mg twice daily), radiological scans showed a near-complete disappearance of lung and lymph node lesions, with significant liver lesion reduction. This case highlights Mebendazole’s potential as a salvage therapy for refractory metastatic colorectal cancer (18).

Lenvatinib, an inhibitor of VEGFRs, was also evaluated for the treatment plan for its ability to address both angiogenesis and the possible VEGF pathway activation due to the TP53 alteration and because of its efficacy reported on AML cell lines (19). Combining anti-angiogenic drugs with immunotherapy has shown promise in enhancing the effectiveness of immunotherapies (20). Extensive literature supports the combination of Lenvatinib with immunotherapy agents, further reinforcing its selection. Research indicates synergistic interactions between these treatments, suggesting that lenvatinib’s anti-angiogenic properties may increase the immune system’s response, resulting in better outcomes for cancer patients (21, 22). Consequently, the evidence supporting the efficacy of lenvatinib and immunotherapy combinations underscores its viability as a treatment option, emphasizing its potential to optimize the efficacy of immunotherapies in clinical settings (23).

The patient’s treatment response was monitored with serial MRD testing and radiological scans. Three weeks post-RFA, MRD was 67.52 MTM/mL. By the fifth month, MRD indicated treatment ineffectiveness with imaging showing tumor growth and new lesions. Radioembolization was then performed.

The MTB subsequently conducted a comprehensive reinvestigation, seeking to identify potential genetic alterations contributing to treatment resistance. Upon obtaining the copy numbers of the amplifications from the test provider Foundation Medicine, a notable finding emerged: the FLT3 amplification exhibited a striking copy number of 62. Given the pivotal role of FLT3 in activating crucial signaling pathways such as Akt, Ras, and Erk, which govern the differentiation, proliferation, and survival of hematopoietic progenitor cells, its aberrant amplification was postulated to underlie the observed resistance to immunotherapy.

Given the rarity of FLT3 amplification in solid tumors, it was not initially prioritized for investigation, especially without specific clinical indicators suggesting its relevance. Furthermore, the oversight was exacerbated by the lack of copy number information immediately following the CGP results. The failure to ascertain this crucial data led to the inadvertent dismissal of FLT3’s exceptionally high copy number.

This incident underscores the critical importance of conducting thorough and comprehensive molecular profiling to guide precision oncology approaches. In retrospect, routine evaluation or specific requests for copy numbers during the initial CGP could have led to the earlier identification of FLT3 amplification in the patient’s treatment management. Early detection of FLT3 amplification could have prompted earlier targeted interventions.

This case highlights the necessity of maintaining a vigilant awareness of rare genomic alterations, even in tumors where they are infrequently reported. It underscores the indispensable role of comprehensive molecular profiling and interdisciplinary molecular tumor boards in ensuring the identification and appropriate consideration of all clinically relevant alterations in the management of cancer patients.

While FLT3 amplifications in CRC are relatively rare, comprising approximately 4% of cases (24), it’s essential to recognize their potential clinical significance within the landscape of targeted alterations. Despite its seemingly low frequency, FLT3 amplification shouldn’t be overlooked, especially considering its broader presence in other solid tumors such as gastric cancers, lung adenocarcinomas, and breast cancers (24). This suggests a potentially significant role for FLT3 amplification not only in CRC but also in targeted therapies across various tumor types.

For comparison, microsatellite instability-high (MSI-H) cases that have a seemingly higher prevalence in clinical practice, are found in 15% of all CRC cases and only 5% in stage IV (25). Despite their lower occurrence, MSI-H cases are routinely screened due to their actionable therapeutic implications. Similarly, although FLT3 amplifications may not be as commonly reported as some other mutations, this broader presence across various tumor types suggests a potentially significant role for FLT3 amplification not only in carcinogenesis but also in targeted therapies despite its relatively low incidence.

FLT3 inhibitors, including midostaurin and sorafenib, were assessed, with sorafenib eventually being prioritized in the treatment plan. This decision was made based on sorafenib’s broader spectrum of activity and its ability to simultaneously target FLT3 and the VEGF pathway which aligns with the patient’s TP53 gene alteration and its potential to trigger angiogenesis and VEGF pathway activation (26). Sorafenib’s immunomodulatory effects and midostaurin’s lack of efficacy in TP53 altered tumors (27) further strengthened its rationale in combination with dual immunotherapy.

The efficacy of sorafenib in targeting FLT3 amplification has been demonstrated in various studies, including a clinical case study involving a rectal adenocarcinoma patient with multiple genetic alterations, including FLT3 amplification. Despite progression on standard irinotecan and oxaliplatin based therapies bevacizumab, and radiotherapy, sorafenib treatment led to rapid clinical improvement in this patient, underscoring its potential as a targeted therapeutic approach (28). Sorafenib’s efficacy on FLT3 alterations was also reported in a study involving six children with relapsed/refractory AML where four patients initially achieved complete remission with sorafenib, three of them experienced relapse within 14 to 37 weeks (29). Interestingly, sunitinib has been identified as a potential option for patients resistant to sorafenib, particularly those harboring secondary FLT3 tyrosine kinase domain mutations. However, in this case, as the patient lacked FLT3 mutations and considering the available combinational data on sorafenib and immunotherapy for hepatocellular carcinoma (HCC) sorafenib was deemed the more suitable choice (30, 31).

The treatment plan was finally adjusted to incorporate Sorafenib and dual immunotherapy with mebendazole. The following MRD assessment, conducted after three months of this modified treatment regimen, demonstrated a drastic reduction to zero, indicating a remarkable therapeutic response. The molecular and radiological complete response is still ongoing at the time of publication, with the patient being closely monitored through routine MRD testing.

## Conclusion

In conclusion, this case exemplifies the successful application of precision oncology principles, guided by an MTB, in managing a CRC patient with a complex mutational profile. The use of comprehensive genomic profiling (CGP) was crucial in tailoring precise and targeted treatment strategies, identifying actionable mutations, and predicting potential resistance mechanisms. A key aspect of the patient’s management was the integration of the MRD testing into the monitoring strategy. The early detection of treatment inefficacy through MRD testing allowed for timely intervention and modification of the treatment plan. This real-time feedback mechanism, coupled with the flexibility inherent in precision oncology, emphasizes the importance of a dynamic and responsive approach in optimizing patient outcomes. Furthermore, this case highlights the educational value that rare and complex cases bring to the MTBs. The ability of the MTB to re-evaluate and adapt, particularly in the face of uncommon molecular alterations like FLT3 amplification in solid tumors, underscores the continual learning process inherent in precision oncology. The knowledge gained from such cases contributes to the ongoing improvement and refinement of treatment strategies for patients with unique molecular profiles. The insights derived from this case not only offer a personalized and effective therapeutic approach for the patient but also offer valuable lessons to the oncology community.

## Data availability statement

The original contributions presented in the study are included in the article/supplementary material. Further inquiries can be directed to the corresponding authors.

## Ethics statement

Ethical approval was not required for patients receiving CGP-based personalized treatment, as it is not mandated by law in our country. Given our focus on advanced or metastatic cancer patients, regulatory requirements for ethical approval do not apply. However, we prioritize transparency and patient autonomy by obtaining written informed consent from each participant before treatment initiation. Additionally, comprehensive CGP curation reports are provided to facilitate patients seeking second opinions from other medical oncologists. All studies complied with local legislation and institutional requirements, with participants providing written informed consent for their involvement in the study. The studies were conducted in accordance with the local legislation and institutional requirements. The participants provided their written informed consent to participate in this study. Written informed consent was obtained from the individual(s) for the publication of any potentially identifiable images or data included in this article.

## Author contributions

EA: Resources, Visualization, Writing – original draft, Writing – review & editing. ÜT: Resources, Writing – original draft, Writing – review & editing. AA: Resources, Writing – original draft, Writing – review & editing. EÖ: Resources, Writing – original draft, Writing – review & editing. ŞB: Resources, Writing – original draft, Writing – review & editing. MD: Project administration, Resources, Supervision, Writing – original draft, Writing – review & editing.
